# Is autoinducer-2 a universal signal for interspecies communication: a comparative genomic and phylogenetic analysis of the synthesis and signal transduction pathways

**DOI:** 10.1186/1471-2148-4-36

**Published:** 2004-09-29

**Authors:** Jibin Sun, Rolf Daniel, Irene Wagner-Döbler, An-Ping Zeng

**Affiliations:** 1 Department of Genome Analysis, GBF – German Research Center for Biotechnology, Braunschweig, Germany; 2 Institute for Microbiology and Genetics, University of Göttingen, Göttingen, Germany; 3 Research Group Microbial Communication, GBF – German Research Center for Biotechnology, Braunschweig, Germany

## Abstract

**Background:**

Quorum sensing is a process of bacterial cell-to-cell communication involving the production and detection of extracellular signaling molecules called autoinducers. Recently, it has been proposed that autoinducer-2 (AI-2), a furanosyl borate diester derived from the recycling of S-adenosyl-homocysteine (SAH) to homocysteine, serves as a universal signal for interspecies communication.

**Results:**

In this study, 138 completed genomes were examined for the genes involved in the synthesis and detection of AI-2. Except for some symbionts and parasites, all organisms have a pathway to recycle SAH, either using a two-step enzymatic conversion by the Pfs and LuxS enzymes or a one-step conversion using SAH-hydrolase (SahH). 51 organisms including most Gamma-, Beta-, and Epsilonproteobacteria, and Firmicutes possess the Pfs-LuxS pathway, while Archaea, Eukarya, Alphaproteobacteria, Actinobacteria and Cyanobacteria prefer the SahH pathway. In all 138 organisms, only the three *Vibrio *strains had strong, bidirectional matches to the periplasmic AI-2 binding protein LuxP and the central signal relay protein LuxU. The initial two-component sensor kinase protein LuxQ, and the terminal response regulator luxO are found in most Proteobacteria, as well as in some Firmicutes, often in several copies.

**Conclusions:**

The genomic analysis indicates that the LuxS enzyme required for AI-2 synthesis is widespread in bacteria, while the periplasmic binding protein LuxP is only present in *Vibrio *strains. Thus, other organisms may either use components different from the AI-2 signal transduction system of *Vibrio *strains to sense the signal of AI-2, or they do not have such a quorum sensing system at all.

## Background

**Figure 1:**
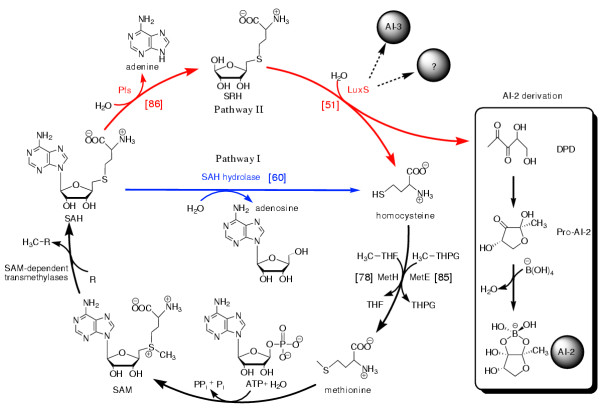
Enzymes involved in the detoxification of SAH and synthesis of AI-2 and AI-3. Abbreviations: SAM, S-adenosyl-methionine; SAH, S-adenosyl-homocysteine; SRH, S-ribosyl-homocysteine; DPD, 4,5-dihydroxyl-2,3-pentanedione; Pro-AI-2, Ai-2 precursor; AI-2, autoinducer 2; AI-3, autoinducer 3. The numbers in brackets show the numbers of analyzed organisms that have that enzyme (reciprocal best hit).

Quorum sensing through small signal molecules called autoinducers is an important process for the regulation of population density dependent cellular processes in bacteria, including the production of antibiotics and virulence factors, conjugation, transformation, swarming behaviour and biofilm formation [1,2]. Recently it was discovered that two different density dependent signal transduction cascades are present in *Vibrio *which converge to trigger luminescence in *V. harveyi *[3] and expression of virulence factors in *V. cholerae *[4,5]. Two chemically different autoinducers are involved in this regulation. While autoinducer-1 is an acylated homoserine lactone (AHL) (N-butanoyl-homoserine lactone) in *V. harveyi*, the structure of autoinducer-2 (AI-2) has been determined in a complex with the sensor protein LuxP [6] and shown to be a furanosyl-borate-diester. It is synthesized in two enzymatic steps (by the Pfs enzyme and the LuxS enzyme) from S-adenosyl-homocysteine (SAH), resulting in 4,5-dihydroxy 2,3 pentanedione (DPD) which undergoes spontaneous cyclization and is then complexed with borate to form AI-2 (Fig. 1).

The LuxS enzyme responsible for the last enzymatic step of AI-2 synthesis is present in a wide phylogenetic range of bacterial genera and AI-2 produced by heterologous organisms triggers luminescence in the *V. harveyi *reporter strains [7,8]. Thus it was hypothesized that AI-2 might be a universal signal molecule. In addition, a large fraction of *E. coli *genes is transcribed differently with culture supernatants containing AI-2 compared to culture supernatants from luxS^- ^mutants [9,10]. Recently it could be shown that the expression of important genes of the virulence islands in *E. coli *serotype O157:H7 (EHEC) is controlled by a LuxS dependent molecule, which was later shown to be not AI-2 but AI-3 whose structure is not known yet and which does not activate the *V. harveyi *bioreporter strain [11]. It is also produced by the gut microflora. Moreover, the host hormone epinephrine activates virulence gene transcription through the same signalling pathway as AI-3, resulting in cross-talk between host and bacterium [11].

Knock-out mutants for luxS have been investigated for modifications of infectious phenotypes in some of the currently sequenced pathogens. In *V. cholerae *[4,5], *Streptococcus pyogenes *[12,13], *Streptococcus pneumoniae *[14], *Neisseeria meningitidis *[15] and *Clostridiuim perfringens *[16] luxS^- ^mutants showed severe defects in the expression of virulence factors. In some other pathogens luxS^- ^mutants showed none or very subtle changes in virulence related traits (e.g. *Borrelia burgdorferi*, [17]; *Porphyromonas gingivalis*, [18]; *Shigella flexneri*, [19]). In *Salmonella typhimurium *AI-2 controls the expression of an ABC transporter which is responsible for the back transport of AI-2 into the cell, presumably to conserve metabolic energy or to interfere with quorum sensing mechanisms of the gut microflora [20].

**Figure 2:**
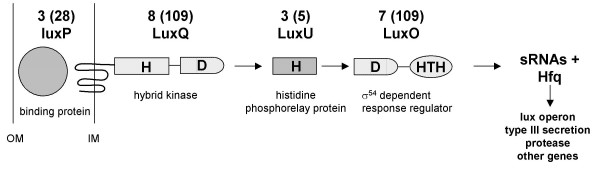
Genes involved in the signalling cascade for detection of AI-2 in *Vibrio*. Abbreviations: OM outer membrane; IM inner membrane; H histidine; D aspartate; HTH helix turn helix motif; sRNA small regulatory RNAs; Hfq chaperone protein. Numbers indicate analyzed organisms that have an orthologous gene for that protein using a reciprocal best hit search strategy, numbers in brackets indicate the number of analysed organisms having a similar gene based on standard blast search.

The signal detection system for AI-2 from *Vibrio *strains is well experimentally proven [4] (Fig. 2). In *V. harveyi *it is composed of a soluble periplasmic AI-2 binding protein LuxP, and a phosphorelay cascade resulting in density dependent activation of the lux operon (Fig. 2). The first step in this cascade is formed by the hybrid sensor kinase LuxQ, which contains both a N-terminal periplasmic membrane bound sensory domain and a C-terminal intracellular response regulator domain [21]. The signal is then transferred to the phosphorelay protein LuxU [22]. This phosphotransferase receives phosphorylation signals both from LuxQ and from LuxN, the parallel, homoserine lactone based quorum sensing circuit of *Vibrio *strains. It phosporylates the final response regulator, LuxO, which belongs to a large, highly conserved family of sigma54 dependent transcriptional regulators. LuxO has three conserved domains, e.g. the response regulator domain, the sigma54 activation domain, and a HTH (helix turn helix) motif for direct DNA binding [23]. At low cell density and in the absence of autoinducers, LuxQ autophosphorylates. The signal is transferred from its conserved aspartate residue to the histidine residue of LuxU, which phorphorylates the aspartate residue of the response regulator LuxO. In its phosphorylated (activated) form, and together with sigma54, LuxO activates the expression of small regulatory RNAs (sRNAs). The complexes of these sRNAs and the sRNA chaperone protein Hfq destabilize the mRNA of the quorum-sensing master regulator LuxR, resulting in the indirect repression of the lux operon transcription [24]. At high cell density, AI-2 present in the periplasmic space binds to the protein LuxP, which converts LuxQ from kinase to phosphatase. This reverses the flow of phosphate through the pathway, from LuxO to LuxU and then to LuxQ. In this case, sRNAs are not expressed. Without destabilization of the sRNA-Hfq complex, LuxR is translated and consequentially the transcription of the Lux operon is switched on.

The LuxS enzyme responsible for the last enzymatic step of AI-2 synthesis has at the same time an important function in the activated methyl cycle of the cell, since it is necessary for recycling of the toxic intermediate SAH [25]. Two pathways are known to be able to degrade and recycle SAH (Fig. 1). One pathway is composed of two successive enzymatic reactions catalysed by LuxS and Pfs. This pathway produces adenine, homocysteine and DPD which can be complexed with borate and converted to AI-2 by two non-enzymatic spontaneous reactions. The other pathway contains only one enzymatic step catalysed by SAH hydrolase (SahH) and produces adenosine and homocysteine. There is no AI-2 production through this pathway. Homocysteine can be further recycled to methionine by MetE or MetH and then activated to SAM by SAM synthetase.

Despite intensive research on AI-2 in the last years the available data do in many cases not allow to clearly separate the metabolic function of the LuxS gene product from its possible signalling activity in interspecies communication. Winzer and his colleagues [25,26] analysed the available genomes (by April 2003) for the presence of LuxS and related genes and critically reviewed the available experimental data with respect to the potential signalling function of AI-2. The emphases of these studies were on the genes pfs, luxS and sahH. A large-scale and more detailed comparative genomic analysis of other genes involved in the AI-2 related metabolic and signal transduction pathways is missing. Therefore, we present here a comprehensive investigation of the phylogenetic distribution of all the genes involved in the synthesis of AI-2, the detoxification of SAH, as well as the signalling cascade necessary for the detection of AI-2 by analysing 138 completely sequenced genomes from the KEGG database [42] and the EMBL database [43]. While LuxS is the enzyme necessary for AI-2 production, it is not required for AI-2 signal transduction. Theoretically, an organism may not be able to produce AI-2 but have the ability to detect the presence of coexisting or competing bacterial species by sensing the environmental concentration of AI-2. This is the case in *Pseudomonas aeruginosa *[27]. Therefore, not only the LuxS-containing organisms but also all other sequenced genomes have been analysed for the existence of the AI-2 signal transduction cascade.

## Results

### Metabolic pathways involved in SAH degradation and recycling

#### SahH and Pfs/LuxS are alternative pathways for recycling of SAH

The distribution of the orthologs of the proteins involved in AI-2 production, SAH degradation and recycling, and AI-2 signalling is listed in Supplementary Table s1 and s2 [additional file 1 and 2]. As shown in Supplementary Table s1 [additional file 1], 80% of the 138 completely sequenced genomes have at least one pathway to degrade SAH. 51 organisms have only the two-step pathway using Pfs and LuxS, while 60 have only the one-step pathway using SahH (Fig. 1). The remaining one-fifth having neither pathway mainly belong to symbionts, intracellular parasites, Mollicutes or Chlamydiates. They probably rely on their host to recycle the toxic intermediate. With the exception of *Bifidobacterium longum *NCCC2705 and *Escherichia blattae*, no organism has both the sahH and luxS gene. Interestingly, each organism has only a single copy of the highly conserved luxS gene. These results are consistent with the studies of Winzer and his colleagues [26].

#### Phylogenetic distribution of SAH detoxification pathways

**Figure 3:**
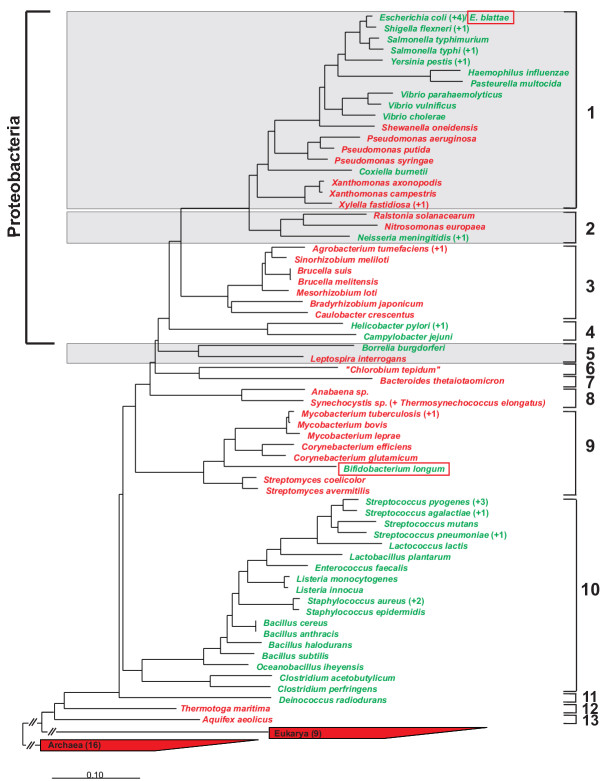
Distribution of one-step (red) and two-step (green) detoxification pathways of SAH within the three domains of life. All sequences shown in the tree could be found in the ARB database (ssujun02.arb), hence were already aligned, and for the desired presentation were all transferred to the rudimentary tree (tree_demo) by the ARB function "quick add by parsimony". After marking all species (sequences) of interest only these were kept by removing all unmarked species with an integrated ARB function. This leaves the original topology of the tree intact while eliminating all species and branches which are unnecessary for the demonstration of the phylogenetic distribution of sequences of prime interest. Phyla are numbered from 1 to 13. 1 Gammaproteobacteria; 2 Betaproteobacteria; 3 Alphaproteobacteria; 4 Epsilonproteobacteria; 5 Spirochaetes; 6 Chlorobia; 7 Bacteroidetes; 8 Cyanobacteria; 9 Actinobacteria; 10 Firmicutes; 11 Deinococcus-Thermus; 12 Thermotogae; 13 Aquificae. Numbers in brackets indicate sequenced genomes analysed. Shaded phyla are mixed, having organisms with the SahH pathway and organisms with the Pfs/LuxS pathway. Boxed strains have both LuxS and SahH.

The presence of either a one-step or a two-step detoxification pathway for SAH follows a phylogenetic pattern. Eukarya and Archaea use exclusively the one-step pathway to degrade SAH, while Bacteria use either the one-step or the two-step pathway depending on their phylogenetic position (Fig. 3). The two-step Pfs/LuxS pathway is consistenly present in all Firmicutes and absent in Actinobacteria (with the exception of *Bifidobacterium longum*). Within the Proteobacteria, Alphaproteobacteria clearly use the one-step detoxification pathway, while there is a dividing line going across the Betaproteobacteria and the Gammaproteobacteria. For the Betaproteobacteria, the pathogen *Neisseria meningitidis *uses the two step pathway, while *Ralstonia solanacearum *and *Nitrosomonas europaea *use the one-step pathway. With the exception of the Xanthomonadales and Pseudomonadales, all Gammaproteobacteria presently sequenced use the Pfs/LuxS pathway.

Only one or two representatives have been sequenced from other microbial phyla, so it is premature to generalize these findings. However, presently the Pfs/LuxS pathway has been found in *Borrelia burgdorferi *(Spirochaetes) and *Deinococcus radiodurans *R1 (Deinococcus-Thermus), while the organisms from other sequenced phyla (Chlorobia, Bacteroidetes, Aquificae, Thermotogae) use the SahH pathway. The second sequenced strain from the phylum Spirochates, *Leptospira interrogans*, uses the SahH pathway. These results are also consistent with the analysis of Winzer et al. [26].

Exploring the ERGO database [44] containing approximately 400 genomes, of which appr. 200 are microbial genomes, resulted in a consistent conclusion on the distribution pattern of SAH hydrolase or Pfs/LuxS degradation pathways (data not shown).

#### Phylogeny of LuxS

**Figure 4:**
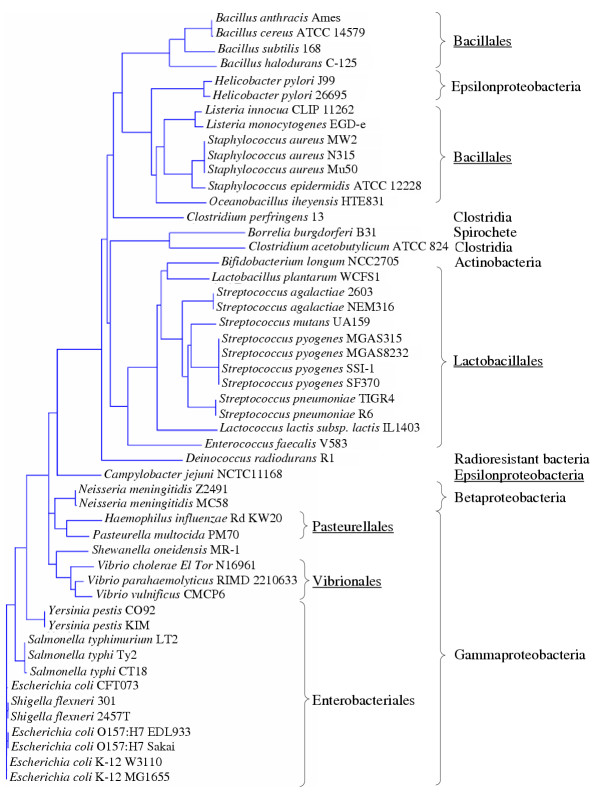
Phylogenetic tree of LuxS proteins from the completely sequenced genomes (138; July 2003) in the KEGG genome database. The tree was constructed using the neighbour-joining (NJ) method after alignment of orthologous genes by means of Vector NTI Advance (InforMax, United States).

The sequences of LuxS orthologs were aligned and a phylogenetic tree was built from the alignment. There are clearly three bigger branches in the phylogenetic tree (Fig. 4). The first contains most Gram negatives, i.e. Gamma- and Betaproteobacteria. The second brach is comprised mainly of Lactobacillales, but contains some other groups as well. Interestingly, the LuxS ortholog from *Bifidobacterium longum*, which is the only species of Actinobacteria having LuxS, and which at the same time has the SahH pathway for recycling of SAH, is most closely related to that of the phylogenetically only distantly related *Lactobacillus plantarum. *Both bacteria share the same habitat, being commensals of the healthy human gut. There would have been ample opportunities for *B. longum *to acquire luxS by horizontal gene transfer from *Lactobacillus*. The Lactobacillus branch also contains a small subcluster with luxS from *Borrelia burgdorferi*, a Spirochete, which is most similar to luxS from *Clostridium acetobutylicum*. The third branch is dominated by Bacillales. Interestingly, it also includes two of three sequenced Epsilonproteobacteria, namely two strains of *Helicobacter pylori*. However, the closely related *Campylobacter jejuni *forms a separate, deeply branching lineage. These data confirm those of Lerat & Moran [28]. Small differences can be attributed to the treeing methods used, e.g. the position of *Campylobacter jejuni *luxS and the fact that γ-Proteobacterial LuxS genes were monophyletic in our analysis, but comprised two different branches in their tree. In addition, we included luxS sequences from *Enterococcus faecalis *and *Deinococcus radiodurans*. The robustness of the tree topology is caused by the high degree of conservation of luxS and strongly supports the resulting conclusions regarding gene transfer for some species.

#### Transformation of homocysteine to methionine

To complete the metabolic cycle of SAH, the common product homocysteine of the two degradative pathways is converted to methionine by a homocysteine methyltransferase (MetE, 5-methyltetrahydropteroyltriglutamate–homocysteine methyltransferase or MetH, 5-methyltetrahydrofolate–homocysteine methyltransferase), then to SAM by SAM synthetase (MetK). SAH is one of the products of SAM-dependent transmethylases. The distribution of MetE, MetH and MetK was analysed in a similar way to LuxS (Supplementary Table s1 [additional file 1]). As a general rule, bacteria that have one of the two pathways to degrade SAH are also able to recycle homocysteine to methionine and to synthesize SAM. This conclusion again supports the importance of the degradation and recycling of SAH. As the only few exceptions, the strains *Helicobacter pylori*, *Streptococcus pyogenes *and *Enterococcus faecalis *lack MetE/MetH but have MetK. The overview of such enzymes in Eukarya and Archaea is more complicated probably because of their incomplete identification by searching homologues to proteins with known functions or because of the existence of other unknown pathways or enzymes in these organisms.

### Signal transduction pathway of AI-2

#### Reciprocal best hit strategy

The reciprocal best-hit orthologs of the signal transduction cascade (LuxP, LuxQ, LuxU and LuxO) from *Vibrio *for the sequenced genomes are listed in Supplementary Table s1 and s2 [additional file 1 and 2]. Unlike the broad distribution of LuxS, the orthologs of the AI-2 binding protein LuxP and the regulator LuxU were found exclusively in *Vibrio *strains. And only *Vibrio *strains have both the orthologs for the hybrid sensor kinase LuxQ and the two component response regulator LuxO. In addition, five other orthologs of LuxQ were found using the reciprocal best hit strategy, namely in *Brucella melitensis*, *Brucella suis*, *Streptococcus agalactiae *(two different strains), and in *Methanosarcina mazei*. For the two component response regulator LuxO, four additional orthologs were found in *Bradyrhizobium japonicum*, *Listeria monocytogenes*, *Lactobacillus plantarum *and *Thermotoga maritima*. Given the complexity of the proteins involved and the limited number of sequences presently available, it is not possible to draw consistent conclusions from this finding at this point.

#### Unidirectional best hit search

However, if not the reciprocal best-hit strategy but the uni-directional best-hit search was applied, 28 organisms were found to have homologues to LuxP. The LuxP homologues of 25 of these organisms were more similar to D-ribose binding proteins of *Vibrio *strains than to the AI-2 binding protein LuxP. We reconstructed the 3D models of LuxP proteins from different *Vibrio *strains by applying the method of SwissModel. All these LuxP proteins have similar 3D structures as expected from the high similarity of their sequences (data not shown). The three-dimensional structure of LuxP is very similar to that of D-ribose-binding proteins [6]. Because of the structural similarity between the ligands (AI-2 and D-ribose), and the structural similarity between the binding proteins, the question has to remain open whether the detected LuxP-homologues in the non-*Vibrio *organisms are actually functional AI-2 binding proteins.

104 organisms were found to have homologues both for the hybrid sensor kinase LuxQ, and the two-component response regulator LuxO. Most organisms had several homologous genes for these two-component systems, so that altogether 315 genes were found for LuxQ and 340 for LuxO. This was caused by the fact that several domains of the sensor and regulator components of signal transduction systems are highly conserved. We were not able to identify the unique binding domain from the sensor protein LuxQ specific for the detection of the AI-2 signal.

## Discussion

### Reciprocal or unidirectional best hit

The reciprocal best hit strategy of sequence similarity comparisons was used here to distinguish orthologous from paralogous genes. Orthologs are genes in different species that evolved from a common ancestral gene by speciation. Paralogs are genes originating from duplication events within a genome. Orthologs tend to retain the same function in the course of evolution, whereas paralogs often evolve new functions [29]. The reciprocal best hit strategy is known to be a better method than the unidirectional best hit method to distinguish orthologs from paralogs [29]. Here, this was especially significant for the identification of components of the AI-2 signal transduction system. The number of identified orthologs for LuxO and LuxQ decreases from over 300 down to 7 and 8 by applying the reciprocal best hit strategy. The resulting orthologs for LuxO and LuxQ are close to the number of identified LuxP and LuxU, confirming that the reciprocal best hit strategy significantly filtered out the potential paralogs.

However, it should be noted that in most cases the functions of these paralogs are not experimentally characterized and cannot be deduced by bioinformatics methods. Because of the high conservation among these regulatory components, it is hard to exclude the possibility that some of them may serve as alternative sensors for detecting the AI-2. On the other hand, the unidirectional best hits for the studied metabolic enzymes were basically the same as the reciprocal best hits, suggesting that the metabolic enzymes for the activated methyl cycle were seldom duplicated during the course of evolution.

### Distribution of LuxS

The presence of either of two possible SAH degradation pathways in most living cells indicates their importance in the central cell metabolism. Both Archaea and Eukarya use exclusively a one-step detoxification pathway for SAH, indicating that this may be the ancient type of metabolism. The distribution of the two-step Pfs/LuxS pathway for detoxification of SAH within the domain Bacteria appears to be phylogenetically conserved. Since the currently sequenced genomes are biased towards pathogens, it remains to be seen if a similar phylogenetic pattern will also be found in non pathogenic bacteria from soils, sediments and marine environments. Previous studies [25,26] came to similar conclusions with respect to the presence of luxS. Our data strengthen the phylogenetic aspect of this distribution.

Interestingly, a unique species from the genomes in the KEGG database, namely *Bifidobacterium longum *NCC2705, possesses both pathways. This species is a key commensal of the healthy human gastrointestinal tract and vagina. The double pathways may be helpful to recycle and use methionine more economically or to accomplish its dependence on H_2_S or methanethiol for methionine biosynthesis [30]. Another non-pathogenic species, *Escherichia blattae*, was also identified to have both pathways (Göttingen Genomics Laboratory, unpublished). However, their physiological roles in this species have still to be clarified.

The phylogenetic tree of LuxS does not in all cases correspond to the 16S rRNA based microbial phylogeny. Thus, horizontal gene transfer might have resulted in the acquisition of LuxS genes e.g. in *Bifidobacterium longum, Helicobacter pylori*, *Clostridium acetobutylicum *and *Borrelia burgdorferi*, with the insect or mammalian gut serving as a melting pot of species.

### Production of AI-2

In most of the microbial genera other than *Vibrio *spp. having a LuxS enzyme the production of AI-2 has been demonstrated using the *Vibrio harveyi *reporter strain BB170 (reviewed by Winzer et al. [26]; otherwise citation is given); e.g. for *Actinobacillus actinomycetemcomitans*, *Bacillus anthracis *[31]; *Borrelia burgdorferi*, *Campylobacter jejuni*, *Clostridium perfringens*, *Escherichia coli*, *Helicobacter pylori*, *Lactobacillus *[32], *Neisseria meningitides*, *Porphyromonas gingivalis*, *Proteus mirabilis*, *Salmonella typhimurium*, *Shigella flexneri*, *Staphylococcus *[27], *Streptococcus*, Pasteurellaceae, periodontal pathogens [33] and rumen bacteria [34]. However, only in *V. harveyi *BB170 it was clearly shown that the active compound was a furanosyl-borate-diester. In *E. coli *serotype O157, Sperandino et al. [11,35-38], showed that the transcription of essential virulence factors coded on the LEE genomic island was triggered by an as yet unknown compound termed autoinducer-3 (AI-3) which depends on the presence of the luxS gene and did not elicit luminescence in *V. harveyi *BB170. Thus, purification of culture supernatants used for detecting AI-2 activity would be required to show that the active fraction is indeed a furanosyl-borate-diester. Conversely, the "real" universal signal might be a different, presently unknown compound produced by a different cyclization product of DPD or by an enzymatic step downstream of LuxS.

### Detection of AI-2

Our data clearly show that the signal transduction cascade for AI-2 is restricted to *Vibrio *species. This is consistent with the results of experimental studies published so far. No alternative signal transduction cascade for AI-2 has been experimentally identified in any of the strains studied, with the exception of *Salmonella typhimurium *[20,39]. Thus, there is no proof that these organisms actually respond to AI-2 in a quorum sensing related manner. However, the large diversity of two-component systems present in these organisms, for which in many cases the specific signals are not known, makes it quite possible that one of them might be devoted to the detection of AI-2 or another LuxS dependent compound. In *Salmonella typhimurium*, an ABC transporter with high homology to ribose transporters, which is however not homologous to LuxP, has been identified whose expression requires the presence of AI-2 and whose function is to transport it back into the cell [20,39]. No AI-2 induced genes other than this transporter have been found, indicating that in this organism AI-2 may not serve as a quorum sensing signal or the appropriate cultivation conditions for the expression of its activity were not met. Orthologs of the *S. typhimurium *lsr genes were found in most Enterobacteriales, as well as in *Sinorhizobium meliloti *and some *Bacillus *sp. (see Supplementary Table s3 [additional file 3]).

However, if AI-2 is indeed a universal signal molecule, it may be useful for bacteria to detect it even if they do not produce it themselves. This was shown to be actually the case in *Pseudomonas aeruginosa*, which does not contain the luxS gene [27]. Here, the promoters of 21 well characterized virulence associated genes were cloned into promoterless *luxCDABE *reporter plasmids and light induction was tested in the presence of AI-2 synthesized enzymatically from SAH or by co-culture with a luxS containing clinical isolate of *Streptotoccus *sp. (strain CF004). The fact that six of these virulence gene promoters were upregulated both by AI-2 and coculture with CF004 suggests a specific effect of AI-2 on the transcription of virulence associated genes in *Pseudomonas aeruginosa*, although the signalling cascade within the cell is presently unknown.

## Conclusions

The presence of luxS in many phylogenetic groups within the domain Bacteria indicates that these bacteria, while recycling SAH in a two-step enzymatic process, at the same time produce a compound able to stimulate luminescence in a *V. harveyi *reporter strain which is most probably a furanosyl-borate-diester. The detection cascade, if any, for this compound in the producing organisms must be different from that in *Vibrio *strains and is presently not known. The diversity of physiological effects observed in luxS^- ^mutants can either be interpreted as the result of a defect in a global quorum sensing regulatory mechanism, which may also be caused by a LuxS dependend compound other than AI-2, or as the result of a defect in the central methyl cycle of the cell. Thus, although there are intriguing indications for a LuxS dependent universal signal molecule in Bacteria, direct proof regarding the chemical nature of the compound and its signalling mechanism in non *Vibrio *organisms is presently missing.

## Methods

### Databases

The protein sequences of 138 sequenced genomes were downloaded from KEGG (Status June 2003) and reformatted as local blast databases. The non-redundant protein database of NCBI (nr) [45] and the KEGG Sequence Similarity Database (SSDB) [46] were explored through their online services.

### Preparation of the queries

To achieve a more complete finding of the proteins functionally similar to the proteins related to either the metabolic pathway (LuxS, Pfs, SahH) or the signal transduction pathway (LuxP, LuxO, LuxQ, LuxU) of autoinducter-2, the NCBI protein database was at first searched with the relevant functional terms such as "AI-2 production" or "LuxS". A phylogenic tree was constructed based on the alignment of the relevant matches by using the component AlignX of the bioinformatic software suite "Vector NTI Advance" (InforMax, United States). From each branch of the tree, one protein (mainly the protein of which the function was manually curated, for example, by SWISSPROT) was selected. All of them were put together into a file as a blast query to represent a function. It is not necessary for the members of this function to be similar to each other in sequence level. This facilitates the finding of evolutionarily far-related proteins by blast search. The protein sequences of MetK, MetE and MetH from *E. coli *K12 and LsrR, -A, -B, -C, -D, -E, -F and G from *Salmonella typhimurium *[39] were used alone as query.

### Blast search

The queries were used to search for their respective orthologs from the local KEGG genome databases by applying the reciprocal best hit strategy [40] with a blastp cutoff E-value 1E-4. In the reciprocal best hit strategy, protein i from genome A is orthologous to protein j from genome B only under the conditions that j is the best hit when i is queried in database B and reciprocally i is also the best hit when j is queried in database A [40]. A Visual Basic script was programmed to realize this strategy automatically. All identified orthologs were submitted for further analysis. The NCBI non-redundant protein database nr was searched using the normal one-direction blastp with a cutoff E-value 1E-4. The hits were manually checked to confirm that they had the same functional annotation as the query.

### Phylogenetic tree construction

The phylogenetic tree for the orthologs was built with the neighbour-joining (NJ) method [41] using Vector NTI Advance (InforMax, United States) after the sequences had been aligned.

## Authors' contributions

JS conducted the data mining work and contributed to writing the manuscript. RD cooperated with the access to the ERGO database. IWD initiated the study, contributed to the concept and drafted the manuscript. APZ supervised the study and contributed to writing the manuscript. All authors read and approved the final manuscript.

## Supplementary Material

Additional File 1 **Supplementary Table s1. **Presence of AI-2 synthesis and detection genes in 138 completed genomes of the KEGG database (July 2003). Abbreviations: luxS, AI-2 synthetase/SRH cleavage enzyme; pfs, SAH-nucleosidase enzyme; sahH, SAH hydrolase; metH and metH, methionine synthetase; metK SAM synthetase; luxP, AI-2 binding protein; luxQ, membrane bound hybrid sensor kinase; luxU, histidine phosphorelay protein ; luxO response regulator. See Fig. 1 for further information on the synthesis pathway and Fig. 2 for the phosporelay detection cascade. Organisms shaded violet contain neither luxS nor sahH. Click here for file

Additional File 2 **Supplementary Table s2. **The accession numbers of the genes in Supplementary Table s1. Abbreviations as in Supplementary Table s1. Click here for file

Additional File 3 **Supplementary Table s3. **Presence of the *Salmonella lsr *gene homologs in the completed genomes of the KEGG database (July 2003). Abbreviations as in Supplementary Table s1. X denotes bi-directional hits, ? denotes unidirectional hits. Click here for file
