# Supplementary material for: Is autoinducer-2 a universal signal for interspecies communication: a comparative genomic and phylogenetic analysis of the synthesis and signal transduction pathways
Source: BMC Evol Biol. 2004 Sep 29;4:36. doi: 10.1186/1471-2148-4-36 (PMC524169; doi:10.1186/1471-2148-4-36)
Supplement: Additional File 1 — Supplementary Table s1. Presence of AI-2 synthesis and detection genes in 138 completed genomes of the KEGG database (July 2003). Abbreviations: luxS, AI-2 synthetase/SRH cleavage enzyme; pfs, SAH-nucleosidase enzyme; sahH, SAH hydrolase; metH and metH, methionine synthetase; metK SAM synthetase; luxP, AI-2 binding protein; luxQ, membrane bound hybrid sensor kinase; luxU, histidine phosphorelay protein ; luxO response regulator. See Fig. 1 for further information on the synthesis pathway and Fig. 2 for the phosporelay detection cascade. Organisms shaded violet contain neither luxS nor sahH. [file 1471-2148-4-36-S1.doc]

**Supplementary Table s1.** Presence of AI-2 synthesis and detection genes in 138 completed genomes of the KEGG database (July 2003). Abbreviations: luxS, AI-2 synthetase/SRH cleavage enzyme; pfs, SAH-nucleosidase enzyme; sahH, SAH hydrolase; metH and metH, methionine synthetase; metK SAM synthetase; luxP, AI-2 binding protein; luxQ, membrane bound hybrid sensor kinase; luxU, histidine phosphorelay protein ; luxO response regulator. See Fig. 1 for further information on the synthesis pathway and Fig. 2 for the phosporelay detection cascade. Organisms shaded violet contain neither luxS nor sahH.

| Category | **Species** | **Abr.** | Genes | | | | | | | | | |
| --- | --- | --- | --- | --- | --- | --- | --- | --- | --- | --- | --- | --- |
|  |  |  | luxS | pfs | sahH | metE | metH | metK | luxP | luxQ | luxO | luxU |
| Eukaryotes | Homo sapiens | hsa |  |  | R |  | R | R |  |  |  |  |
| Mammals | *Mus musculus* | mmu |  |  | R |  |  | R |  |  |  |  |
|  | *Rattus norvegicus* | rno |  |  | R |  | R |  |  |  |  |  |
| Fish | *Danio rerio* | dre |  |  |  |  |  |  |  |  |  |  |
| Insect | *Drosophila melanogaster* | dme |  |  | R |  | R |  |  |  |  |  |
| Nematode | *Caenorhabditis elegans* | cel |  |  | R |  | R |  |  |  |  |  |
| Plants | *Arabidopsis thaliana* | ath |  | R | R | R | R | R |  | U | U |  |
|  | *Oryza sativa japonica* | osa |  |  |  |  | R |  |  | U |  |  |
| Protozoa | *Plasmodium falciparum* | pfa |  |  | R |  |  | R |  |  |  |  |
|  | *Trypanosoma brucei* | tbr |  |  |  |  |  |  |  |  |  |  |
| Fungi | *Saccharomyces cerevisiae* | sce |  |  | R | R | R | R |  | U | U |  |
|  | *Schizosaccharomyces pombe* | spo |  |  | R | R |  |  |  | U |  |  |
|  | *Encephalitozoon cuniculi* | ecu |  |  |  |  |  |  |  |  |  |  |
| **Bacteria** | Escherichia coli *K-12 MG1655* | eco | R | R |  | R | R | R | U | U | U |  |
| Proteobacteria  Gamma-  proteobacteria  Enterobacteriales | *Escherichia coli* K-12 W3110 | ecj | R | R |  | R | R | R | U | U | U |  |
|  | *Escherichia coli* O157:H7 EDL933 | ece | R | R |  | R | R | R | U | U | U |  |
|  | *Escherichia coli* O157:H7 Sakai | ecs | R | R |  | R | R | R | U | U | U |  |
|  | *Escherichia coli* CFT073 | ecc | R | R |  | R | R | R | U | U | U |  |
|  | *Salmonella typhi* CT18 | sty | R | R |  | R | R | R | U | U | U |  |
|  | *Salmonella typhi* Ty2 | stt | R | R |  | R | R | R | U | U | U |  |
|  | *Salmonella typhimurium* LT2 | stm | R | R |  | R | R | R | U | U | U |  |
|  | *Yersinia pestis* CO92 | ype | R | R |  | R | R | R |  | U | U |  |
|  | *Yersinia pestis* KIM | ypk | R | R |  | R | R | R |  | U | U |  |
|  | *Shigella flexneri* 301 (serotype 2a) | sfl | R | R |  | R | R | R |  | U | U |  |
|  | *Shigella flexneri* 2457T (serotype 2a) | sfx | R | R |  | R | R | R |  | U | U |  |
|  | *Buchnera sp.* AP*S (symbiont of Acyrthosiphon pisum)* | buc |  | R |  | R |  | R |  |  |  |  |
|  | *Buchnera aphidicola* Sg (symbiont *Schizaphis graminum)* | bas |  | R |  | R |  | R |  |  |  |  |
|  | *Buchnera aphidicola* (symbiont of *Baizongia pistaciae*) | bab |  | R |  | R |  |  |  |  |  |  |
|  | *Wigglesworthia brevipalpis* | wbr |  | R |  |  |  | R |  | U | U |  |
| Pasteurellales | *Haemophilus influenzae* Rd | hin | R | R |  | R |  | R | U | U | U |  |
|  | *Pasteurella multocida* PM70 | pmu | R | R |  | R |  | R | U | U | U |  |
| Xanthomonadales | *Xylella fastidiosa* 9a5c | xfa |  |  | R | R |  | R |  | U | U |  |
|  | *Xylella fastidiosa Temecula1* | xft |  |  | R | R |  | R |  | U | U |  |
|  | *Xanthomonas campestris pv. campestris* ATCC 33913 | xcc |  |  | R | R | R | R |  | U | U | U |
|  | *Xanthomonas axonopodis* pv. citri 306 | xac |  |  | R | R | R | R |  | U | U | U |
| Vibrionales | *Vibrio cholerae* El Tor N16961 (serotype O1) | vch | R | R |  | R | R | R | R | R | R | R |
|  | *Vibrio vulnificus* CMCP6 | vvu | R | R |  | R | R | R | R | R | R | R |
|  | *Vibrio parahaemolyticus* RIMD 2210633 | vpa | R | R |  | R | R | R | R | R | R | R |
| Pseudomonadales | *Pseudomonas aeruginosa* PA01 | pae |  |  | R | R | R | R |  | U | U |  |
|  | *Pseudomonas putida* KT2440 | ppu |  | R |  | R | R | R |  | U | U |  |
|  | *Pseudomonas syringae* pv. tomato DC3000 | pst |  |  | R | R | R | R | U | U | U |  |
| Legionellales | *Coxiella burnetii* SA 493 | cbu |  |  | R | R |  | R |  | U | U |  |
| Alteromonadaceae | *Shewanella oneidensis* MR-1 | son | R | R |  | R | R | R |  | U | U |  |
| Beta-proteobacteria | *Neisseria meningitidis* MC58 (serogroup B) | nme | R | R |  | R |  | R |  | U | U |  |
|  | *Neisseria meningitidis* Z2491 (serogroup A) | nma | R | R |  | R |  | R |  | U | U |  |
|  | *Ralstonia solanacearum* GMI1000 | rso |  | R | R | R | R | R |  | U | U |  |
|  | Nitrosomonas europaea *ATCC 19718* | neu |  |  | R | R | R | R |  | U | U |  |
| Epsilon-proteobacteria | *Helicobacter pylori* 26695 | hpy | R | R |  |  |  | R |  | U | U |  |
|  | *Helicobacter pylori* J99 | hpj | R | R |  |  |  | R |  | U | U |  |
|  | *Campylobacter jejuni* NCTC11168 | cje | R | R |  | R |  | R |  | U | U |  |
| Alpha-proteobacteria | *Rickettsia prowazekii* Madrid E | rpr |  |  |  |  |  |  |  | U | U |  |
|  | *Rickettsia conorii* Malish 7 | rco |  |  |  |  |  |  |  | U | U |  |
|  | *Mesorhizobium loti* MAFF303099 | mlo |  | R | R | R | R | R |  | U | U |  |
|  | *Sinorhizobium meliloti* 1021 | sme |  | R | R |  | R | R | U | U | U |  |
|  | *Agrobacterium tumefaciens* C58 (UWash/Dupont) | atu |  | R | R | R | R | R | U | U | U |  |
|  | *Agrobacterium tumefaciens* C58 (Cereon) | atc |  | R | R | R | R | R | U | U | U |  |
|  | *Brucella melitensis* 16M | bme |  | R | R |  | R | R |  | R | U |  |
|  | *Brucella suis* 1330 | bms |  | R | R |  | R | R |  | R | U |  |
|  | *Bradyrhizobium japonicum* USDA110 | bja |  | R | R | R | R | R |  | U | R |  |
|  | *Caulobacter crescentus* | ccr |  | R | R | R | R | R |  | U | U |  |
| Firmicutes | *Bacillus subtilis* 168 | bsu | R | R |  | R | R | R | U | U | U |  |
| Bacillales | *Bacillus halodurans* C-125 | bha | R | R |  | R | R | R | U | U | U |  |
|  | *Bacillus anthracis* Ames | ban | R | R |  | R | R | R |  | U | U |  |
|  | *Bacillus cereus* ATCC 14579 | bce | R | R |  | R | R | R |  | U | U |  |
|  | *Oceanobacillus iheyensis* HTE831 | oih | R | R |  |  | R | R | U | U | U |  |
|  | *Staphylococcus aureus* N315 (meticillin-resistant) | sau | R | R |  | R | R | R |  | U | U |  |
|  | *Staphylococcus aureus* Mu50 (vancomycin-resistant) | sav | R | R |  | R | R | R |  | U | U |  |
|  | *Staphylococcus aureus* MW2 | sam | R | R |  | R | R | R |  | U | U |  |
|  | *Staphylococcus epidermidis* ATCC 12228 | sep | R | R |  | R | R | R |  | U | U |  |
|  | *Listeria monocytogenes* EGD-e | lmo | R | R |  | R | R | R |  | U | R |  |
|  | *Listeria innocua* CLIP 11262 | lin | R | R |  | R | R | R |  | U | U |  |
| Lactobacillales | *Lactococcus lactis* IL1403 | lla | R | R |  | R |  | R |  | U | U |  |
|  | *Streptococcus pyogenes* SF370 (serotype M1) | spy | R | R |  |  |  | R |  | U | U |  |
|  | *Streptococcus pyogenes* MGAS8232 (serotype M18) | spm | R | R |  |  |  | R |  | U | U |  |
|  | *Streptococcus pyogenes* MGAS315 (serotype M3) | spg | R | R |  |  |  | R |  | U | U |  |
|  | *Streptococcus pyogenes* SSI-1 (serotype M3) | sps | R | R |  |  |  | R |  | U | U |  |
|  | *Streptococcus pneumoniae* TIGR4 | spn | R | R |  | R |  | R |  | U | U |  |
|  | *Streptococcus pneumoniae* R6 | spr | R | R |  | R |  | R |  | U | U |  |
|  | *Streptococcus agalactiae* 2603 (serotype V) | sag | R | R |  | R | R | R | U | R | U |  |
|  | *Streptococcus agalactiae* NEM316 | san | R | R |  | R | R | R | U | R | U |  |
|  | *Streptococcus mutans* UA159 (serotype C) | smu | R | R |  | R | R | R |  | U | U |  |
|  | *Lactobacillus plantarum* WCFS1 | lpl | R | R |  | R | R | R |  | U | R |  |
|  | *Enterococcus faecalis* V583 | efa | R | R |  |  |  | R |  | U | U |  |
| Clostridia | *Clostridium acetobutylicum* ATCC824 | cac | R | R |  |  | R | R |  | U | U |  |
|  | *Clostridium perfringens* 13 | cpe | R | R |  |  | R | R | U | U | U |  |
|  | *Clostridium tetani* E88 | ctc |  | R |  |  | R | R | U | U | U |  |
|  | *Thermoanaerobacter tengcongensis* MB4T | tte |  |  |  |  | R | R | U | U | U |  |
| Mollicutes | *Mycoplasma genitalium* G-37 | mge |  |  |  |  |  | R |  |  |  |  |
|  | *Mycoplasma pneumoniae* M129 | mpn |  |  |  |  |  | R |  |  |  |  |
|  | *Mycoplasma pulmonis* | mpu |  |  |  |  |  | R |  |  |  |  |
|  | *Mycoplasma penetrans* HF-2 | mpe |  | R |  |  |  | R |  |  |  |  |
|  | *Mycoplasma gallisepticum* R | mga |  | R |  |  |  | R |  |  |  |  |
|  | *Ureaplasma urealyticum* (serovar 3) | uur |  | R |  |  |  | R |  |  |  |  |
| Actinobacteria | *Mycobacterium tuberculosis* H37Rv (lab strain) | mtu |  | R | R | R | R | R |  | U | U |  |
|  | *Mycobacterium tuberculosis* CDC1551 | mtc |  | R | R | R | R | R |  | U | U |  |
|  | *Mycobacterium bovis* AF2122/97 | mbo |  | R | R | R | R | R |  | U | U |  |
|  | *Mycobacterium leprae* TN | mle |  |  | R | R | R | R |  | U | U |  |
|  | *Corynebacterium glutamicum* ATCC 13032 | cgl |  | R | R | R | R | R | U | U | U |  |
|  | *Corynebacterium efficiens* YS-314 | cef |  | R | R | R | R | R |  | U | U |  |
|  | *Streptomyces coelicolor* A3(2) | sco |  | R | R | R | R | R | U | U | U |  |
|  | *Streptomyces avermitilis* | sma |  | R | R | R | R | R |  | U | U |  |
|  | *Bifidobacterium longum* NCC2705 | blo | R | R | R | R |  | R |  | U | U |  |
|  | *Tropheryma whipplei* Twist | twh |  |  |  | R |  | R |  | U | U |  |
|  | *Tropheryma whipplei* TW08/27 | tws |  |  |  | R |  | R |  | U | U |  |
| Fusobacteria | *Fusobacterium nucleatum* ATCC 25586 | fnu |  | R |  |  | R | R |  | U | U |  |
| Chlamydia | *Chlamydia trachomatis* (serovar D) | ctr |  |  |  |  |  |  |  | U | U |  |
|  | *Chlamydia muridarum* (*Chlamydia trachomatis* MoPn) | cmu |  |  |  |  |  |  |  | U | U |  |
|  | *Chlamydophila pneumoniae* CWL029 | cpn |  | R |  |  |  |  |  | U | U |  |
|  | *Chlamydophila pneumoniae* AR39 | cpa |  | R |  |  |  |  |  | U | U |  |
|  | *Chlamydophila pneumoniae* J138 | cpj |  | R |  |  |  |  |  | U | U |  |
|  | *Chlamydophila caviae* GPIC | cca |  | R |  |  |  |  |  | U | U |  |
| Spirochete | *Borrelia burgdorferi* B31 | bbu | R | R |  |  |  | R |  | U | U |  |
|  | *Treponema pallidum* Nichols | tpa |  | R |  |  |  | R |  |  | U |  |
|  | *Leptospira interrogans* 56601 (serovar lai) | lil |  |  | R |  | R | R |  | U | U |  |
| Bacteroid | *Bacteroides thetaiotaomicron* VPI-5482 | bth |  | R | R |  | R | R |  | U | U |  |
| Cyanobacteria | *Synechocystis sp.* PCC6803 | syn |  |  | R |  | R | R |  | U | U |  |
|  | *Thermosynechococcus elongatus* BP-1 | tel |  |  | R | R | R | R |  | U | U |  |
|  | *Anabaena sp.* PCC7120 *(Nostoc sp.* PCC7120*)* | ana |  |  | R |  | R | R |  | U | U |  |
| Green sulfur bac. | *Chlorobium tepidum* TLS | cte |  |  | R |  | R | R |  | U | U |  |
| Hyperthermophilic bacteria | *Aquifex aeolicus* VF5 | aae |  |  | R | R |  | R |  | U | U |  |
|  | *Thermotoga maritima* MSB8 | tma |  | R | R | R | R | R | U | U | R |  |
| Radioresistant bac. | *Deinococcus radiodurans* R1 | dra | R | R |  |  | R | R |  | U | U |  |
| Archaea | *Methanococcus jannaschii* DSM2661 | mja |  |  | R | R |  |  |  |  |  |  |
| Euryarchaeota | *Methanosarcina acetivorans* C2A | mac |  | R | R |  | R |  |  | U | U |  |
|  | *Methanosarcina mazei* Goe1 | mma |  |  | R |  | R |  |  | R | U |  |
|  | *Methanobacterium thermoautotrophicum* deltaH | mth |  |  | R | R | R |  |  | U | U |  |
|  | *Methanopyrus kandleri* AV19 | mka |  |  | R | R |  | R |  |  |  |  |
|  | *Archaeoglobus fulgidus* DSM4304 | afu |  |  | R |  | R |  |  | U | U |  |
|  | *Halobacterium sp.* NRC-1 | hal |  |  | R |  |  | R |  | U | U |  |
|  | Thermoplasma acidophilum | tac |  |  | R | R |  | R |  |  |  |  |
|  | *Thermoplasma* *volcanium* GSS1 | tvo |  |  | R | R |  | R |  |  |  |  |
|  | *Pyrococcus* *horikoshii* OT3 | pho |  |  | R | R |  | R |  | U | U |  |
|  | *Pyrococcus* *abyssi* | pab |  |  | R | R |  | R |  |  | U |  |
|  | *Pyrococcus* *furiosus* DSM3638 | pfu |  |  | R | R |  | R |  |  |  |  |
| Crenarchaeota | *Aeropyrum* *pernix* K1 | ape |  |  | R | R |  |  |  |  |  |  |
|  | *Sulfolobus* *solfataricus* P2 | sso |  |  | R | R |  |  |  |  |  |  |
|  | *Sulfolobus* *tokodaii* strain7 | sto |  |  | R | R |  |  |  |  |  |  |
|  | *Pyrobaculum* *aerophilum* IM2 | pai |  |  | R | R |  |  |  |  |  |  |
| **Sum** |  | **138** | 51 | 86 | 60 | 85 | 78 | 112 | 3(28) | 8(109) | 7(109) | 3(5) |

1. “R” means the presence of a reciprocal-best-hit orthologous gene in a specific genome. “U” means only uni-directional hit. The cutoff E-value is 1E-4.
2. The presence of luxS, pfs is marked as green background while the one of sahH as red.
3. The genomes which have neither luxS nor sahH are marked as violet background.
